# Innovations in the quantitative virus outgrowth assay and its use in clinical trials

**DOI:** 10.1186/s12977-017-0381-2

**Published:** 2017-12-21

**Authors:** Nicholas J. Norton, Axel Fun, Mikaila Bandara, Mark R. Wills, Hoi Ping Mok, Andrew M. L. Lever

**Affiliations:** 1 Department of Medicine, University of Cambridge, Cambridge, UK; 2 Yong Loo Lin School of Medicine, National University of Singapore, Singapore, Singapore

**Keywords:** HIV, Latency, Viral outgrowth assay

## Abstract

A robust measure of the size of the latent HIV reservoir is essential to quantifying the effect of interventions designed to deplete the pool of reactivatable, replication competent proviruses. In addition to the ability to measure a biologically relevant parameter, any assay designed to be used in a clinical trial needs to be reproducible and scalable. The need to quantify the number of resting CD4+ T cells capable of releasing infectious virus has led to the development of the quantitative viral outgrowth assay (VOA). The assay as originally described has a number of features that limit its scalability for use in clinical trials; however recent developments reducing the time and manpower requirements of the assay, while importantly improving reproducibility mean that it is becoming much more practical for it to enter into more widespread use. This review describes the background to VOA development and the practical issues that they present in utilising them in clinical trials. It describes the innovations that have made their usage more practical and the limitations that still exist.

## Background

### Development of the VOA

The possibility of a reservoir of latently infected CD4+ T cells harbouring proviral DNA but not producing virus was identified in early clinical studies on peripheral blood from HIV infected persons [1]. When antiretroviral therapy later transformed the prognosis for people living with infection it became clear that this reservoir of latently infected cells would be a major barrier to eradicating the virus. The number of resting CD4+ T cells harbouring replication competent viruses has been considered the most accurate measure of the latent pool as it is thought that these cells are the source of the virus which rebounds after cessation of antiviral therapy [2, 3]. The demonstration that a population of CD4+ HLA-DR- T cells from patients on effective treatment could be made to release virus upon stimulation with phytohaemagglutinin (PHA) led to efforts to quantify the latent reservoir [4–6]. These studies established the principle of quantitation by seeding resting CD4+ T cells in limiting dilution before activating the cells with a combination of PHA and irradiated allogeneic peripheral blood mononuclear cells (PBMCs). Due to the low frequency of latently infected cells it was necessary to obtain a large volume blood sample (approximately 200 ml) from study participants.

The amount of HIV Gag protein released by reactivating a single latently infected cell is below the limit of detection of a standard p24 ELISA. Therefore the released virus is amplified by co-culture with permissive cells. In the original assay the cells were co-cultured with PHA stimulated, CD8-depleted PBMCs from an HIV-negative donor. Maximum likelihood statistics became the standard to estimate the frequency of latently infected resting CD4+ T cells, expressed as infectious units per million cells (IUPM) [7].

The use of allogeneic CD8-depleted PBMCs to amplify outgrowth virus has biological and practical limitations. PBMCs from different donors are not equally permissive for HIV infection, leading to different readouts depending on the PBMC donor. This problem is particularly acute when amplifying R5 tropic virus as expression levels of CCR5 vary widely between donors, CXCR4 on the other hand tends to be highly expressed making detection of X4 tropic virus less problematic. One solution is pre-screening of donor PBMCs for CCR5 expression level and selecting for high CCR5-expressing donors only. Another option is using cells from matched donor-patient pairs that are shown in vitro to exhibit robust viral replication, a method that also requires extensive pre-screening prior to the actual assay [8]. Although donor matching reduces inter assay variability for samples obtained from the same HIV-positive donor, it makes the procedure more labour intensive; and together with the need to perform leukapheresis to obtain sufficient latently infected cells, makes it harder for VOA to scale up to meet the needs of a clinical trial.

## Developments of VOA for clinical trial use

### Novel VOA

A number of modifications have been introduced to improve the original viral outgrowth assay. Two new methods utilise cell lines engineered to express high levels of CCR5 [9, 10]. These cell line based assays have been shown to give IUPM readings similar to PBMC based assays [9, 10] and to give highly consistent results when sampling the same patient repeatedly [10]. Cell line based systems are also less labour intensive as the cells do not have to be freshly prepared from blood and stimulated with PHA for each assay. One assay uses a custom antibody cocktail to purify resting CD4+ T cells from PBMCs of HIV positive donors in a single step, significantly reducing processing time from approximately 2 h to 45 min [10], a huge advantage when scaling up assay numbers for clinical trials. Another recent modification proposed to reduce the work required for each assay is stimulating the resting CD4+ T cells with CD3/CD28 microbeads rather than PHA and irradiated allogeneic PBMCs. Although the results correlated well with those derived from the standard stimulus, the microbead based method appeared to be less sensitive [11].

### Read-outs

**Fig. 1:**
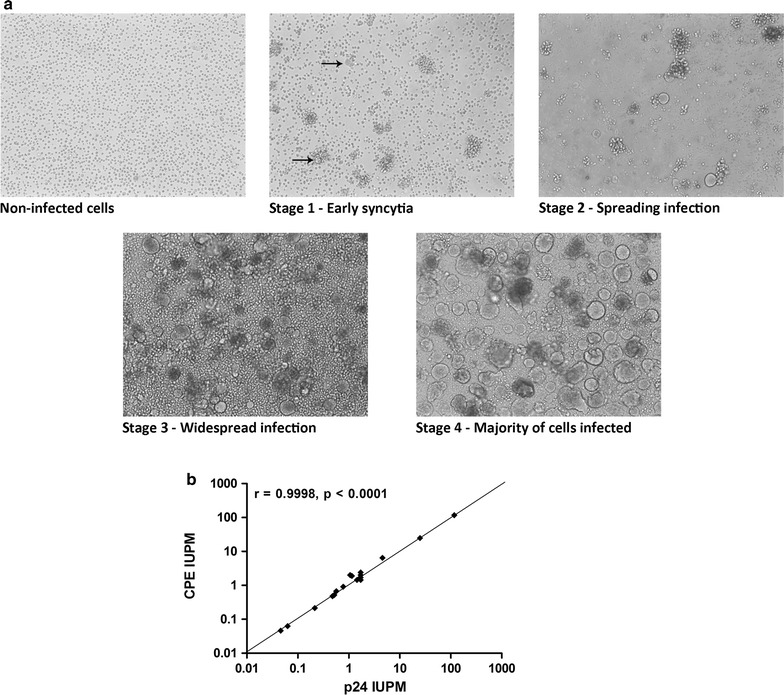
**a** HIV infected SupT1-CCR5 cells form syncytia which can be readily discerned by microscopy. **b** The results of VOA using observed cytopathic effect (CPE) as readouts strongly correlate with that using p24 ELISA

Recently a number of strategies have been proposed for optimising the outgrowth assay by improving the sensitivity of the detection system or simplifying the read-out. Besides substituting the PBMC based co-culture with a CD4/CXCR4/CCR5 expressing clonal cell line, we recently reported that the observation of viral cytopathic effect in these cells showed a near-perfect correlation with the detection of p24 from infected cells (Fig. 1) [10], further simplifying the assay. Alternatively, use of a more sensitive PCR based detection method instead of the p24 ELISA can reduce the co-culture time required to detect replication-competent HIV [9]. The TILDA assay [12] utilises the detection of an increase in multiply spliced HIV RNA in CD4+ T cells after stimulation. Similar to the VOA, the cells are seeded in limiting dilution to obtain a quantitative measure. It uses total rather than resting CD4+ T cells, which has the advantage of requiring a smaller blood volume than a VOA and it does not need an extended period of culture for detection, however this means that it measures the inducible reservoir in a population of cells different to that used in the VOA. Another new assay, TZA, utilizes TZM-bl cells [13]. These cells produce β-galactosidase in response to viral Tat, enabling the presence of HIV to be determined by the enzymatic cleavage of a luminescent compound. In contrast to TILDA the TZA assay utilises resting CD4+ T cells but also requires a significantly smaller blood volume than the standard VOA. Neither of these assays correlated closely with IUPM measured by a standard VOA implying that they may not measure the same component of the HIV reservoir. Another caveat is that as neither assay requires viral replication to detect reactivated virus, neither provides unequivocal proof that the detected viral products are derived from replication-competent virus.

Another novel technique that significantly shortens the assay time of the VOA, by use of an enhanced sensitivity of p24 detection at ultra-low concentrations, has recently been applied to HIV latency research [14]. This ultrasensitive assay utilises Simoa single molecule automated array technology [15] which can measure sub-femtomolar concentrations of analytes using a micro-bead based antibody capture system. This promising method allows the detection and quantitation of p24 produced by a single reactivated infected CD4+ T cell [14]. Although this method is very powerful in detecting viral protein production very early after reactivation, it is not yet clear if the measure represents replication-competent virus and how it correlates to the VOA. Therefore, the results of ongoing clinical studies like RIVER (Clinical trial registry identifier: NCT02336074) which employ both a VOA and the ultrasensitive p24 assay, among many other measures of the latent HIV reservoir, will be of tremendous interest in the search for a definitive measure, or combination of measures, of the clinically significant HIV reservoir.

As efforts to cure HIV progress it may become necessary to be able to detect smaller reservoirs with greater confidence. A recent development on the VOA has been the development of the murine outgrowth system [16–18]. It involves the injection of PBMCs or CD4 T cells into immunodeficient NSG mice [17, 18] or humanised mice [16]. These systems offer enhanced sensitivity as they are capable of sampling large number of cells. They can also give a semi-quantitative estimate of the reservoir size and outgrowth from patient cells where a standard VOA had been negative [16]. However, these assays are not easily scalable, limiting their deployment.

### Clinical utility

VOA is generally regarded as the gold standard in estimating the size of the replication-competent latent viral reservoir. Interestingly in the REDUC trial [19], combinatorial application of a histone deacetylase inhibitor romidepsin, an HIV vaccine, and recombinant human GM-CSF has achieved a statistically significant reduction on the size of the latent reservoir as measured by total HIV DNA and by VOA in resting CD4 T cells, but not by integrated HIV DNA in total CD4 T cells. This illustrates the importance of including a range of outcome measures in clinical trials aimed at perturbing the latent reservoir until a definitive measure is identified. A number of other clinical studies have also included VOA as outcomes (Table 1). These studies represent only a fraction of the cure effort and a recent survey [20] shows that VOA is being deployed in a only a minority (5 out of 25) of ongoing clinical trials on HIV cure. This highlights how the logistic difficulties outlined above and the associated high costs limit the applicability of the assay.

**Table 1:** Examples of clinical trials using VOA

Clinical trial no.	Intervention	Key finding	References
NCT01319383	Vorinostat (single dose) 8 patients	Single dose of vorinostat increased cell-associated RNA in resting CD4 cells but no reduction in IUPM	[26]
NCT01319383	Vorinostat (multiple doses 3 ×/week for 8 weeks) 5 patients	Increase in cell-associated RNA with multiple doses compared to single dose of vorinostat in 3/5 patients, no reduction in IUPM	[27]
NCT01319383	Vorinostat (multiple doses) 16 patients	Multiple doses of vorinostat increased cell-associated RNA in resting CD4 cells but no reduction in IUPM	[28]
NCT01680094	Panobinostat 15 patients	Increase in cell-associated RNA, increase in plasma viraemia, no significant reduction in total and integrated DNA or IUPM. No delay in time to rebound in the 9 patients undergoing planned treatment interruption.	[29]
NCT00289952	Valproic acid and ART 56 patients	No reduction in IUPM	[30]
NCT01286259	Disulfiram 16 patients	Transient increase in plasma viraemia in a subset of patients, no overall change in plasma viraemia in the entire cohort, no change in IUPM	[31]
NCT02443935	MGN1703 15 patients	Significant reduction in cell-associated RNA post-treatment, transient increase in plasma viraemia in 6/15 patients. No change in total or integrated HIV DNA, also no change in IUPM	[32]
NCT02092116 REDUC	Romidepsin, Vacc-4x and rhuGM-CSF 20 patients (17 completed trial)	Significant decrease (40%) in total DNA (in 16 patients measurable), non-significant decrease (19%) in integrated DNA (15 patients measurable) significant reduction (38%) in IUPM (6 patients measurable). No delay in time to rebound in the 15 patients who have undergone post treatment interruption	[19]
NCT02336074 RIVER	ART, Raltegravir, Vorinostat, ChAdV63.HIVconsv and MVA.HIVconsv	Ongoing	
NCT02707900 VORVAX	Vorinostat and AGS-004	Ongoing	
NCT02471430 ACTIVATE	Panobinostat and Pegylated Interferon-alpha2a	Ongoing	
NCT02408861	Nivolumab and Ipilimumab	Ongoing	

*IUPM* infectious units per million cells as measured by VOA

## Conclusions

Although there have been significant improvements in VOAs enhancing their applicability problems remain. Assays of the same patient performed in different laboratories can yield different estimates of reservoir size, in part due to different experimental protocols. As yet there has been no international consensus on the optimum protocol for measuring the latent reservoir, nor have there been efforts to standardise readouts using samples of known latent viral load. Addressing these issues will be essential to enable comparison of the results of clinical trials carried out at different centres. Additional limitations of the VOA stem from the biology of HIV latency. Thus far, no method has been able to activate HIV in all latently infected cells, for which repeated rounds of reactivation may be necessary [21, 22]. It is not known whether the stimuli we have available are capable of reactivating every provirus as our understanding of latency is incomplete and there is emerging evidence of a diversity of mechanisms responsible for viral silencing [23]. Furthermore, cells carrying replication-competent latent HIV are so rare that large volume blood draws, or leukapheresis, may remain a requisite for any VOA, adding to the logistic challenges of deploying and scaling up the assay. The pathophysiological importance of the parameter measured by VOA is the most compelling reason for it still to be considered a sine qua non of clinical trials of virus reactivation. However, as with all assays based on sampling of blood, it does not sample latently infected T cells in the tissue compartment. It is unclear if the latent T cells reservoir in the blood compartment fully recapitulate that in the tissue compartment, as tissue resident T memory cells do not circulate [24]. Critically, there are as yet no robust data on how VOA correlates with the time to viral rebound after withdrawal of ART, which is ultimately the clinically relevant outcome. It is sobering to note that the ‘Mississippi child’ had a undetectable latent viral load after cumulative sampling of 64 million resting CD4+ T cells [25] prior to rebound. Thus, whilst VOA is a powerful tool as it gives a physiologically relevant readout in clinical trials evaluating the efficacy of latency reversal, developing a simpler and cheaper but still reproducible assay for the latent reservoir and establishing predictive parameters for clinical outcomes remain urgent research priorities.
